# Environmental resistance development to influenza antivirals: a case exemplifying the need for a multidisciplinary One Health approach including physicians

**DOI:** 10.1186/s13028-018-0360-1

**Published:** 2018-01-25

**Authors:** Josef D. Järhult

**Affiliations:** Zoonosis Science Center, Department of Medical Sciences, Uppsala University, 75185 Uppsala, Sweden

**Keywords:** Avian influenza, Drug residue, Influenza A virus, Lanamivir, Mallard, Neuraminidase inhibitor, Oseltamivir, Pandemic preparedness, Peramivir, Zanamivir

## Abstract

A multidisciplinary approach is a prerequisite for One Health. Physicians are important players in the One Health team, yet they are often hard to convince of the benefits of the One Health approach. Here, the case for multidisciplinarity including physicians is made using the example of environmental resistance development to influenza antivirals. Neuraminidase inhibitors are the major class of anti-influenza pharmaceuticals, and extensively stockpiled globally as a cornerstone of pandemic preparedness, especially important in the first phase before vaccines can be mass-produced. The active metabolite of oseltamivir that is excreted from treated patients degrades poorly in conventional sewage treatment processes and has been found in river waters. Dabbling ducks constitute the natural influenza A virus reservoir and often reside near sewage treatment plant outlets, where they may be exposed to neuraminidase inhibitor residues. In vivo experiments using influenza-infected Mallards exposed to neuraminidase inhibitors present in their water have shown resistance development and persistence, demonstrating that resistance may be induced and become established in the influenza strains circulating in natural hosts. Neuraminidase inhibitor resistance genes may become part of a human-adapted influenza virus with pandemic potential through reassortment or direct transmission. A pandemic caused by a neuraminidase inhibitor-resistant influenza virus is a serious threat as the first line defense in pandemic preparedness would be disarmed. To assess the risk for environmental influenza resistance development, a broad multidisciplinary team containing chemists, social scientists, veterinarians, biologists, ecologists, virologists, epidemiologists, and physicians is needed. Information about One Health early in high school and undergraduate training, an active participation of One Health-engaged physicians in the debate, and more One Health-adapted funding and publication possibilities are suggested to increase the possibility to engage physicians.

## Introduction

Although the One Health approach has recently gained increasing traction, engaging physicians remains a challenge. Whereas veterinarians generally have a thorough understanding of the association of animal and human health—introduced already at an early stage of their training—physicians tend to struggle appreciating humans as yet another animal species and tend to have an overly anthropocentric view. A multitude of professionals need to work together in One Health and are equally important; the multidisciplinarity per se is an important feature of the One Health concept. Yet, engaging specifically physicians in One Health issues is important for several reasons such as: (1) their expertise is needed to plan and evaluate projects from a human health perspective; (2) the engagement of physicians is needed to underscore and give credibility to the human health impact of the issue in question; (3) engagement of physicians can help bring the issue in question to the attention of policy makers and the general public; and (4) engagement of physicians can help open doors to funding aimed primarily at human health. Likely, engaging physicians in One Health is a long, multi-step process that involves interventions at an early phase of their training as well as making already practicing physicians aware of the concept and its importance. In this literature review, environmental resistance development to influenza antivirals will be used as an example to illustrate the importance of multidisciplinarity and engagement of physicians.

## Influenza in the avian-human interface

Influenza A virus (IAV) is a pathogen with major economic and health implications in both human and veterinary medicine. Stamping out interventions cost animal lives and cause huge economic losses to the poultry industry, and seasonal as well as pandemic outbreaks in humans strain health care budgets and organizations. Though important to human health, IAV is a zoonotic virus; dabbling ducks and other waterfowl constitute the natural reservoir [1, 2]. Occasional spill-over events occur to other species, humans included. Thus, genetic material from avian IAVs is the basis for human IAVs. The transfer of IAV genetic material from avian sources to humans can occur through two entirely different routes, *direct transmission* and *reassortment*. Direct transmission means that an avian IAV infects humans without previous adaptation in a non-avian host. This is exemplified by transmission of highly-pathogenic avian influenza viruses (HPAIVs) such as H5N1 from infected poultry to humans. Reassortment on the other hand occurs when two or more IAVs infect the same host cell simultaneously. There is no proofing mechanism to sort genetic segments from the respective parental IAV strain into coherent viral offspring, and thus reassortants containing all different variations of the gene segments from each parental strain will be formed. In the context of avian-to-human spread, reassortment is mostly a slow and stepwise process involving several reassortment events over time. As an example, the 2009 pandemic H1N1 IAV was formed from genetic segments from three different IAVs circulating in swine, initially originating from avian sources 1918–1998 and formed through multiple reassortment events [3].

## The potential for environmental resistance development in the natural influenza host

**Fig. 1:**
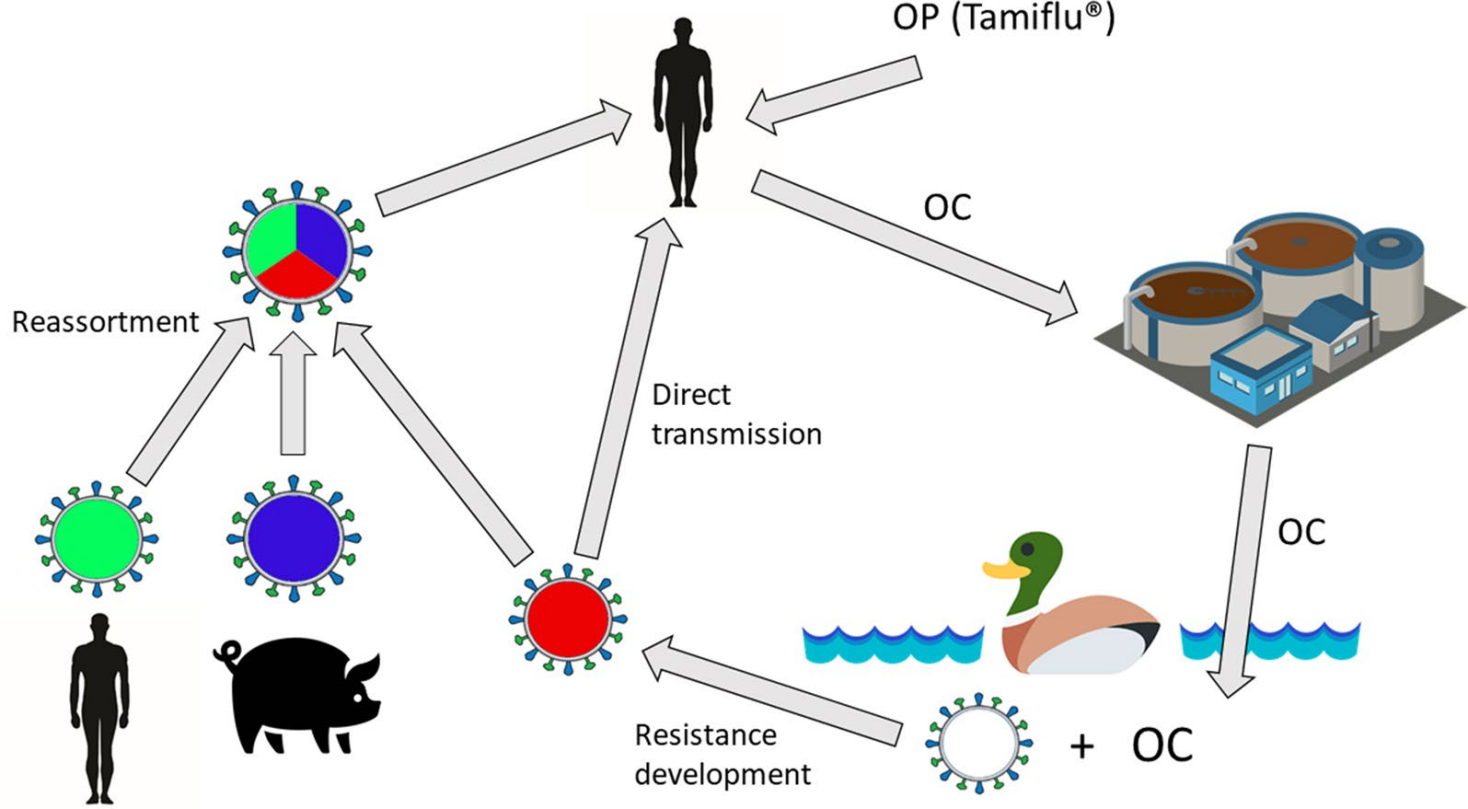
As OC degrades poorly in STPs and surface waters, it can enter aquatic environments where dabbling ducks can be exposed to the substance. Dabbling ducks constitute the natural influenza reservoir and have a perpetual circulation of influenza A virus in their population. Thus, there is a risk of resistance development in the intestine of the ducks where replicating virus and OC co-exist. Through reassortment or direct transmission, an oseltamivir-resistant influenza virus could spread to humans. *OC* oseltamivir carboxylate, *OP* oseltamivir phosphate, *STP* sewage treatment plant

Given the connection of IAVs in the avian reservoir and in humans, resistance development to antiviral drugs in naturally circulating avian IAVs is a potential concern also for human health. Pandemic preparedness plans rely heavily on antivirals in the first phase before vaccines can be mass-produced, and antivirals are stockpiled extensively [4]. Experiences from the 2009 IAV pandemic demonstrate that the timely global production and distribution of vaccines was even more difficult to achieve that previously estimated [5]. Thus, antiviral drugs play a crucial role in the beginning of a new IAV outbreak, regardless if the origin is reassortment (like the last four pandemics) or direct transmission (e.g. a human-adapted HPAIV). To date, neuraminidase inhibitors (NAIs) constitute the absolute majority of anti-influenza drugs used. The other, older class of anti-influenza antivirals on the market, adamantanes, are largely abandoned due to side effects and resistance development [6]. The most used NAI, oseltamivir (Tamiflu ©), is administered as a prodrug, oseltamivir phosphate, and rapidly converted in the human body to oseltamivir carboxylate (OC), the active metabolite. OC is excreted mainly via urine and remains stable in surface water and sewage treatment processes [7]. Thus, there is a risk that discharge of OC from sewage treatment plants (STPs) pollutes water bodies downstream of the STP outlets. Dabbling ducks such as the Mallard constitute the natural influenza reservoir, and often reside in waters downstream of STPs. Therefore, dabbling ducks may be exposed to OC in their water environment. IAV is a gastrointestinal infection in Mallards [8] and hence replicating IAV and low levels of OC could co-exist in the intestine of the Mallard, creating a risk for resistance development. If OC-resistant- or NAI-resistant-strains are established among IAVs circulating in the natural reservoir, resistance could be an inherent property of newly human-introduced IAVs, either through reassortment or direct transmission. This is a worrisome scenario given the crucial importance of NAIs in pandemic preparedness. The environmental resistance development hypothesis and its potential connection to humans is depicted in Fig. 1, and has also previously been reviewed [9, 10].

## Occurrence of neuraminidase inhibitors in the environment

Ample evidence has accumulated to demonstrate the poor degradation of NAIs in STPs, and the occurrence of NAIs in the environment. OC has been demonstrated in effluent water from STPs [11], as have the newer NAIs zanamivir (Relenza©) [12], peramivir [13], and lanamivir [13]. All four NAIs have also been detected in river water; OC up to 865 ng/L [12, 14], zanamivir 59 ng/L [12, 15], peramivir 11 ng/L [13], and lanamivir 9 ng/L [13]. The highest NAI levels have been found in Japan, the top world-wide per-capita consumer, but OC has also been found in river waters in Europe, e.g. in the UK up to 193 ng/L [16]. One study has also highlighted discharge from drug production facilities as a potential contributing factor to environmental pollution of oseltamivir [17]. To analyze degradation and presence of antiviral drugs in the environment, as well as assessing the implications, environmental chemists are vital in understanding and combatting environmental IAV resistance.

A prerequisite for the occurrence of NAIs in the environment is that the drugs are being used. In most parts of the world, use is regulated through prescription by physicians. Thus, involvement of physicians in a One Health approach, enabling them to appreciate the risks with NAI prescription in a broader perspective, is important to obtain a prudent use of NAIs. Multiple studies, many of them drug company-sponsored, have failed to demonstrate effects of oseltamivir and zanamivir (the second most used NAI) on uncomplicated influenza in otherwise healthy patients than simply shortening of length of clinical disease (symptoms) by 1 day (e.g. [18]). Hence, liberal use of NAIs to for uncomplicated influenza can be questioned, and should definitely be avoided if symptoms have been present > 48 h before treatment, as the effect of NAIs is much dependent of early start of treatment. To implement these guidelines, participation of other health professionals such as nurses and medical practitioners is important. A special case is NAI use in parts of the world where antiviral drugs are sold over the counter (without prescription). Under these circumstances, self-medication with NAIs without previous medical consultation is likely a major driver for NAI pollution. Thus, educating the general public about One Health, as well as strengthening local health systems are important measures in this setting. Social science professionals, e.g. behavioral scientists, are especially important to help understand prescriptions/drug use in a cultural context.

## Resistance development in LPAIVs infecting Mallards exposed to NAIs

As NAIs are present in river water, what is the risk of IAV resistance development in the natural reservoir? Mallards perpetuate low pathogenic IAVs (LPAIVs) with a pronounced spatial and temporal prevalence variation; in the Northern Hemisphere the prevalence is typically high (up to 60%) during fall migration and low (0.4–2%) at wintering grounds [19]. Several in vivo studies using LPAIV-infected Mallards subjected to low levels of OC in their water have demonstrated resistance development. Exposure of a H1N1 LPAIV to 0.95 µg/L of OC resulted in the well-known resistance mutation H275Y [20], H5N2 exposure to 1 µg/L in E199V [21], H6N2–12 µg/L in R292K [22], and H7N9–2.5 µg/L in I222T [23]. At least for the H1H1 and H5N2 IAVs, detected OC levels in river water are of the same magnitude as where resistance development occurred. Similar in vivo Mallard studies addressing the risk of resistance development to other NAIs are important, especially as these drugs may be more widely used in the future in case of oseltamivir resistance. To assess the risk for resistance development, several players in the One Health team are needed; bird ecologists to understand migration patterns and behavior of dabbling ducks, virologists to elucidate resistance development at a molecular and functional level, and veterinarians to investigate the aspect of influenza disease in birds.

## Persistence of resistance without drug pressure

Once resistance is induced among LPAIVs circulating among wild birds, it is imperative to assess if the resistance can persist without drug pressure. Influenza outbreaks are sporadic, and thus NAIs are not constantly present in the environment. Further, the NAI resistance dogma has been that resistance can quite easily develop during treatment but that resistance development is of less concern, as mutants have decreased fitness—as noted in e.g. early in vitro oseltamivir studies [24]. However, a human seasonal H1N1 IAV strain resistant to oseltamivir through the H275Y mutation spread globally during the 2007–2009 influenza seasons, and the spread was not correlated to oseltamivir use [25]. This demonstrated that in certain genetic backgrounds, NAI resistance do not cause decreased IAV fitness. Several compensatory mutations likely contributed to the ability of the seasonal H1N1 IAV to harbor H275Y without fitness loss [26, 27]. Interestingly, Mallard in vivo experiments demonstrate that in a H1N1 LPAIV that acquired H275Y when infected Mallards were subjected to OC in their water [20], resistance persisted despite removal of OC from the water of the Mallards and subsequent IAV replication and transmission [28]. On the contrary, in a H6N2 virus harboring the R292K mutation from an in vivo experiment [22], resistance did not persist without drug pressure [29], illustrating the impact of different IAV genetic backgrounds. Thus, both from human epidemiological data and in vivo studies, there is evidence that in certain genetic backgrounds, IAV resistance mutations do not result in decreased viral fitness. Here, public health/epidemiology expertise is important to successfully assess spread of resistant strains in the human population and the relation to NAI use. Clinical pharmacists can contribute to NAI prescription analysis and physicians can provide expertise in human influenza disease and drug use from a prescriber’s perspective.

## Risk of resistance transmission to humans

### Reassortment

If resistance to NAIs can develop in IAVs circulating in the natural host, and in certain genetic backgrounds persist without drug pressure, what is the risk that the resistant NA gene becomes part of a human IAV? All four pandemic IAVs seen during the last century were formed through reassortment and all of them were formed by genetic material of avian origin [30, 31]. Thus, it is possible that a NAI-resistant NA gene originating from the natural host can form part of a new pandemic IAV through reassortment. However several factors can influence the likelihood of this event: (1) How prevalent are NAI-resistance among IAVs circulating in the natural host? (2) Is there a loss of fitness when the NAI-resistant NA gene reassorts with other IAVs, i.e. is there a barrier for reassortment? (3) For how long is the NAI-resistant IAV circulating in other hosts (e.g. swine) before it spills over to humans, i.e. what delay is there from resistance development in the environment/natural host until human introduction? To start answering these questions several actions and professionals are needed – such as IAV surveillance in wild waterbirds by biologists and experimental studies regarding reassortment including a NAI-resistant NA gene by virologists. To keep the research questions linked to the human health perspective, involvement of physicians is important.

### Direct transmission

There is a barrier for direct transmission of avian-adapted IAVs to humans. The virus must overcome several hurdles such as differences in host body temperature, receptor architecture, and immune response. To date, this has precluded efficient human-to-human transmission of directly transmitted IAVs. However, two research groups have found that small changes in a H5N1 HPAIV allowed for mammal-to-mammal transmission [32, 33]. In one of the studies five point mutations were sufficient to enable transmission, and a subsequent study demonstrated that some circulating H5N1 strains already carried two out of the five point mutations and has modelled factors that can increase the probability for acquisition of the last three [34]. Thus, the genetic barrier for direct transmission may not be as protective as previously thought, and given the high morbidity and mortality for H5N1 and H7N9 IAVs [35], sustained human-to-human transmission is a serious threat. NAI-resistance in such an IAV would make matters much worse; preparedness plans initially rely on stockpiles of NAIs and resistance could render them useless. To assess the risks for human health and to guide pandemic preparedness planning, physicians are crucial. Other important players include virologists to assess the genetic barrier for direct transmission in different IAV genetic backgrounds and environmental settings, immunologists to provide knowledge of human and avian immune defenses and their differences, and professionals with skills in logistics and societal structure to implement the findings into pandemic preparedness.

**Fig. 2:**
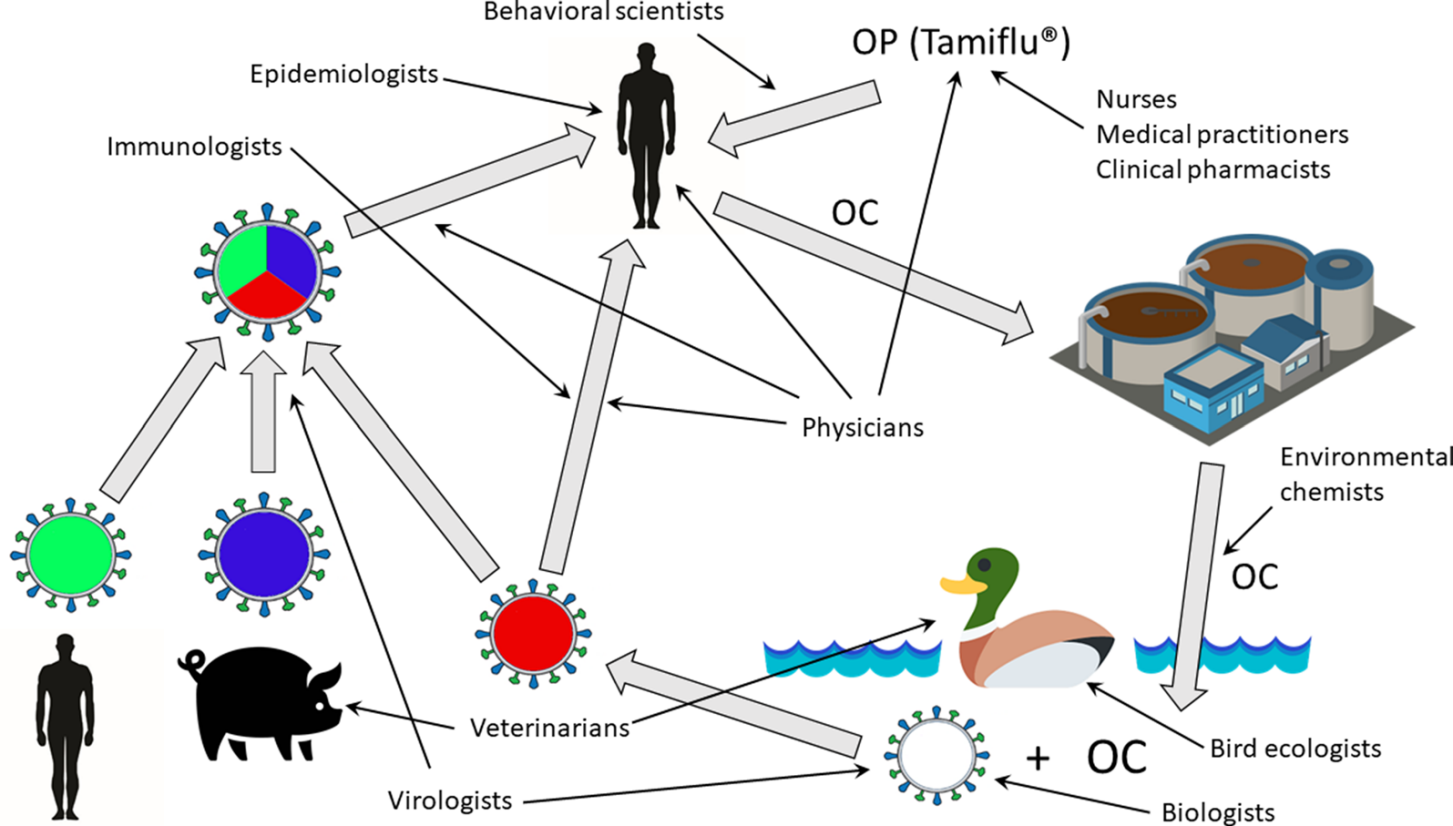
An example of key players in the One Health team needed to successfully tackle NAI resistance development in the environment

A summary of key players in a One Health team investigating environmental resistance development to influenza antivirals is depicted in Fig. 2.

## Conclusions

Multidisciplinarity is a key element of the One Health approach, and it is imperative to engage physicians as one of several key players in One Health questions. The example of environmental resistance development in influenza demonstrates this, but it is true for most other One Health questions as well. Engaging physicians in One Health is a challenging task—it is the author’s opinion that information and discussion activities early in high school and undergraduate training, a more active voice of One Health-engaged physicians, and funding and publication possibilities more suited to One Health research are important factors in the process.

## Abbreviations

HPAIV: highly pathogenic avian influenza virus
IAV: influenza A virus
LPAIV: low-pathogenic avian influenza virus
NA: neuraminidase
NAI: neuraminidase inhibitor
OC: oseltamivir carboxylate
STP: sewage treatment plant
